# Additives Type Schiff’s Base as Modifiers of the Optical Response in Holographic Polymer-Dispersed Liquid Crystals

**DOI:** 10.3390/polym9070298

**Published:** 2017-07-21

**Authors:** Sandra Fenoll, Víctor Navarro-Fuster, Manuel Ortuño, Jose Luis Serrano, Andrés Márquez, Sergi Gallego, Inmaculada Pascual, Augusto Beléndez

**Affiliations:** 1 Instituto Universitario de Física Aplicada a las Ciencias y las Tecnologías, Universidad de Alicante. Apartado 99, 03080 Alicante, Spain; fenoll.sandra@gmail.com (S.F.); Victor.Navarro@ua.es (V.N.-F.); andres.marquez@ua.es (A.M.); sergi.gallego@ua.es (S.G.); pascual@ua.es (I.P.); a.belendez@ua.es (A.B.); 2 Departamento de Física, Ingeniería de Sistemas y Teoría de la Señal, Universidad de Alicante, Apartado 99, E03080 Alicante, Spain; 3 Departamento de Óptica, Farmacología y Anatomía, Universidad de Alicante, Apartado 99, E03080 Alicante, Spain; 4 Departamento de Ingeniería Minera, Geológica y Cartográfica, Universidad Politécnica de Cartagena, Área de Química Inorgánica, Regional Campus of International Excellence ‘Campus Mare Nostrum’, 30203 Cartagena, Spain; jose.serrano@upct.es

**Keywords:** holography, photopolymers, liquid crystals, Schiff’s base, HPDLC

## Abstract

Schiff’s bases with specific π-electron system have been synthesized and used as additives in holographic polymer-dispersed liquid crystals. It was observed that these substances modify different parameters such as current intensity, voltage, and diffracted light intensity. In addition, the maximum diffraction efficiency obtained in the reconstruction of the holograms is related to the additive molecule. We propose a relationship between this behavior and the molecular structure of these substances.

## 1. Introduction

The incorporation of liquid crystals (LCs) in photopolymers makes possible to obtain composite materials which can modify their optical properties by means of an electric field. The LC molecules add optical anisotropy to the photopolymer, and therefore it is possible to change the response by modifying the electric field applied [1,2]. 

Holographic polymer-dispersed liquid crystals are known as H-PDLCs. They are made by holographic recording in a photo-polymerization induced phase separation process (PIPS) in which the liquid crystal molecules diffuse to dark zones in the diffraction grating where they can be oriented by means of an electric field. The orientation of the liquid crystal produces a refractive index variation which changes the diffraction efficiency. Therefore, the grating develops a dynamic behavior that can be modified by means of an electronic device [3,4,5]. 

H-PDLC composites have a high degree of complexity because they combine the characteristics of the liquid crystals with the chemistry and polymerization reactions of the photopolymers. From the perspective of holography, it is not a simple recording material because they are devices that combine optics with electronics and photochemistry with nanotechnology. This makes it possible for H-PDLCs to be studied from many different points of view [6,7].

One focus of study is based on the improvement of the performance of the devices, and also on the introduction of substances that modify their behaviour [8,9,10].

In this work, substances known as Schiff’s bases have been synthesized and used as additives in a holographic photopolymer containing nematic liquid crystal in order to study their possible influence in the optical response of the material [11]. We have designed these substances taking into account a size and molecular structure with a delocalized π-electron system similar to those of the liquid crystal molecule. Moreover, we have added different substituents to the molecule, and this introduces mesomeric and inductive effects which changes the behavior of the electronic delocalization. We study whether these substances have an influence on the interaction of the liquid crystal molecules with the external electric field applied to the H-PDLC device.

## 2. Photopolymer Formulation

We used a photopolymer formulation studied by our research group in previous works [12,13]. The monomer used was dipentaerythritol penta/hexa-acrylate (DPHPA) with a refractive index *n* = 1.490. We used the nematic liquid crystal QYPDLC-036 (LC) from Qingdao QY Liquid Crystal Co., Ltd., Qingdao, China. which is a mixture of 4-cyanobiphenyls with alkyl chains of different lengths. It has an ordinary refractive index *n*_0_ = 1.520, and a difference between extraordinary and ordinary index Δ*n* = 0.250. *N*-methyl-2-pyrrolidone (NMP) was used as solvent. Octanoic acid (OA) was used as cosolvent and surfactant. We also used ethyl eosin (YEt) as dye and *N*-phenyl glycine (NPG) as initiator. Table 1 shows the composition of the photopolymer.

The solution was made by mixing the components under red light, where the material is not sensitive. Modified photopolymer was prepared by adding 4 mg of the Schiff’s base additive (synthesized in Section 2.1) to 300 μL of solution. The mix was mechanically stirred, sonicated for 10 min at 35 °C in an ultrasonic bath, deposited between two 1 mm-thick indium tin oxide (ITO) conductive glass plates and separated with non-calibrated glass hollow microspheres from Aldrich with an average diameter of 13 µm.

### 2.1. Chemical Synthesis of the Additives: Modifier A, B, and C

**Scheme 1:**
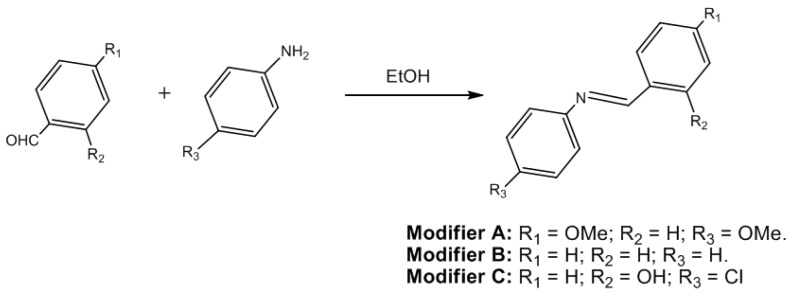
Synthesis of Schiff base ligands.

We made three different substances: modifiers A, B, and C (Scheme 1) by chemical synthesis. Their molecular structure was designed to obtain electron delocalization in the molecule. These substances are Schiff’s base ligands—A family of organic compounds named after Hugo Schiff [11]. These are formed when any primary amine reacts with an aldehyde or a ketone under specific conditions. Thus, a Schiff’s base can be considered as a nitrogen analogue of an aldehyde or ketone in which the carbonyl group has been replaced by an imine or azomethine group.

The history and relevance of Schiff’s bases has recently been reviewed, including the different synthetic approaches developed to-date [14]. We have adapted a previously reported [15] procedure of synthesis as follows: A solution of the corresponding aniline (*p*-anisidine for A; aniline for B; *p*-chloroaniline for C) (10.9 mmol) in ethanol (5 mL) was added slowly to a vigorously stirred solution of the respective aldehyde (*p*-anisaldehyde for A; benzaldehyde for B; salicylaldehyde for C). The solution changed colour instantly, and after stirring for 1 h at room temperature the obtained precipitate was collected by filtration, washed with a small amount of ethanol, and vacuum dried. The products thus obtained were purified upon recrystallization from ethanol, and are displayed in Scheme 1.

**Figure 1:**
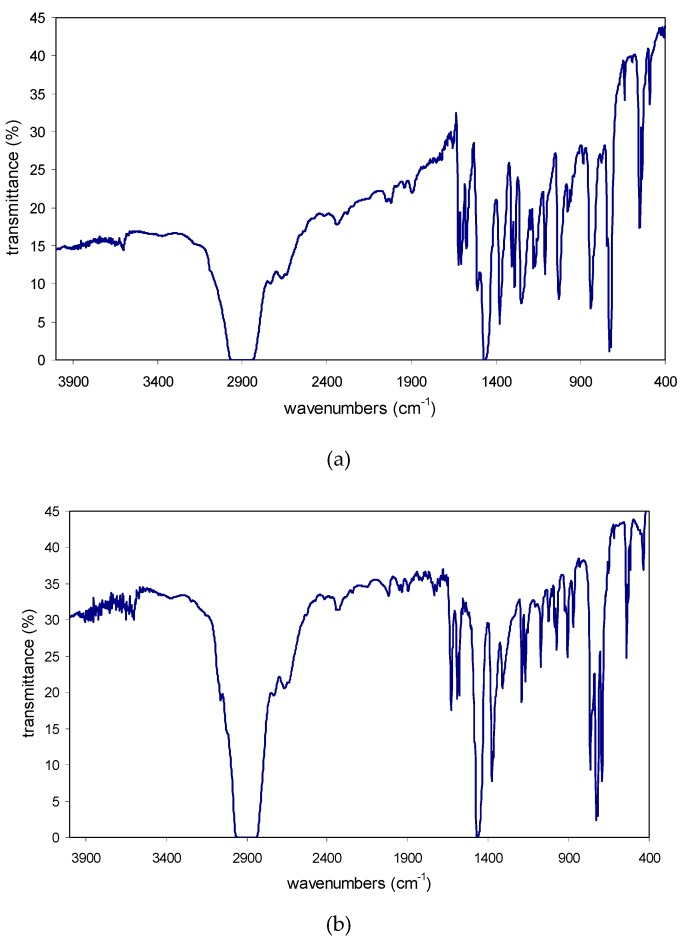

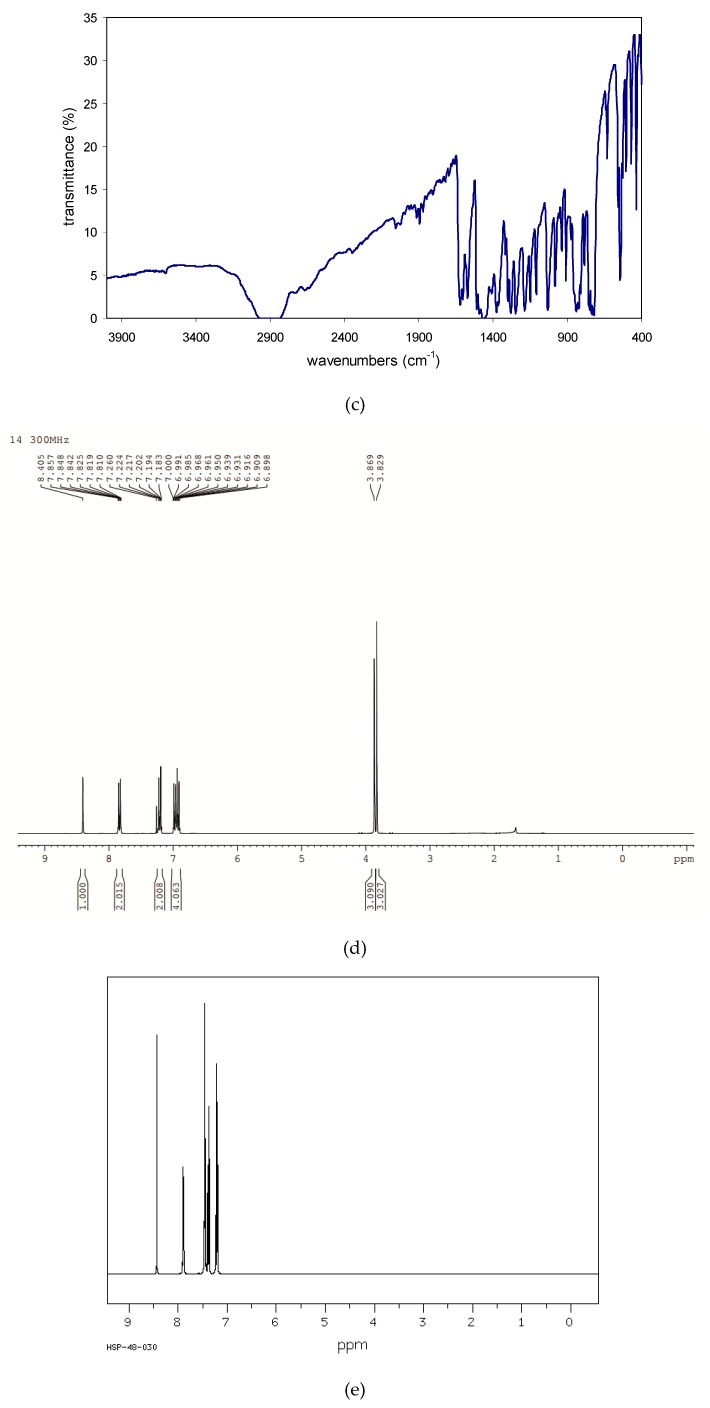

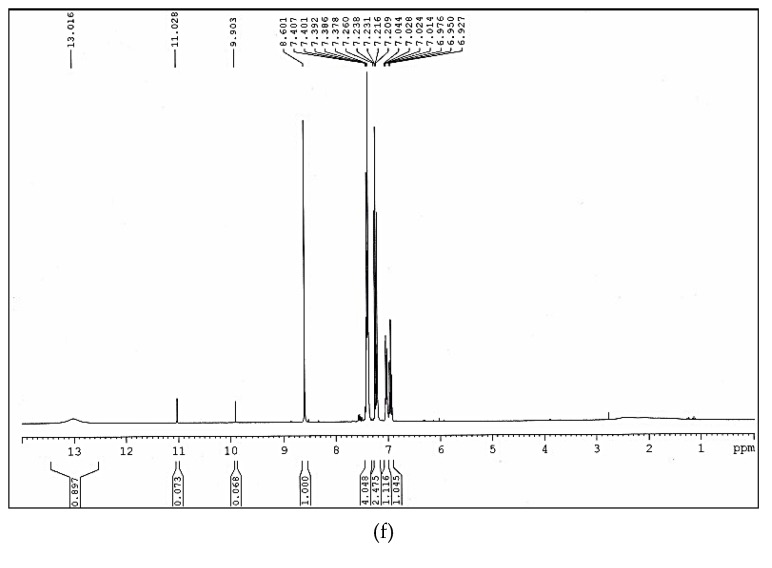
Fourier transform infrared spectroscopy (FT-IR) (**a**–**c**) and proton nuclear magnetic resonance (^1^H NMR) spectra (**d**–**f**) for modifiers A, B, and C.

The synthesized modifiers A, B, and C were characterized by Fourier transform infrared spectroscopy (FT-IR) and proton nuclear magnetic resonance (^1^H NMR). The FT-IR and ^1^H NMR spectra are included in Figure 1. 

*N*-(4-Methoxybenzylidene)-4-methoxyaniline (A): (2.07 g, 77%), IR (cm^−1^): 1625 m; 1604 m; 1579 m; 1509 m, 1308 m; 1290 s; 1250 s; 1183 m, 1113 m; 1027 s; 970 m; 842 s, 634 m; 552 s; 497 m. ^1^H NMR (200 MHz, CDCl_3_): 8.40 (s, 1H, N=CH), 7.83 (m, 2H), 7.20 (m, 2H), 6.95 (m, 4H), 3.87 (s, 3H, CH_3_O), 3.83 (s, 3H, CH_3_O). Anal. calc. for C_15_H_15_NO_2_: C 74.67; H, 6.27; N, 5.81; Found: C, 74.75; H, 6.30; N, 6.90.

*N*-Benzylideneaniline (B): (1.68 g, 85%), IR (cm^−1^): 1631 s; 1585 s; 1314 m; 1198 s; 1174 m; 1076 m; 976 m; 909 m; 765 m; 549 s; 442 m. ^1^H NMR (200 MHz, CDCl_3_): 8.43 (s, 1H, N=CH), 7.89 (m, 2H), 7.46 (m, 3H), 7.38 (m, 2H), 7.22 (m, 1H), 7.20 (m, 2H). Anal. calc. for C_13_H_11_N; C, 86.15; H, 6.12; N, 7.73. Found: C, 86.35; H, 6.13; N, 7.77.

*N*-(salicilydeneden)-4-chloroaniline (C): (2.35 g, 93%), IR (cm^−1^): 1613 s; 1588 m; 1280 s; 1180 s, 1152 s; 1095 s; 1030 m; 1012 m, 960 m; 845 s; 760 s; 695 m, 564 m; 518 s; 448 m; 421 m. ^1^NMR (200 MHz, CDCl_3_): 13.01 (s, 1H, OH), 8.60 (s, 1H, N=CH), 7.39 (m, 4H), 7.23 (m, 2H), 7.03 (m, 2H). Anal. calc. for: C_14_H_12_ClN C, 73.20; H, 5.27; N, 6.10; Found: C, 73.31; H, 5.30; N, 6.19.

Typical iminic absorbances in the region 1700–1600 cm^−1^ and the absence of free aldehyde/amine bands indicate that condensation took place neatly. Two strong bands around 1000 and 850 cm^−1^ in the spectrum of modifier A are characteristic of para-substituted aromatic rings. Regarding ^1^H NMR, both chemical shifts and integration of signals are in accordance with the proposed structure. Low field resonance of the iminic proton at 8.40 ppm and the presence of two different MeO– groups in the aliphatic region are clearly observed in Figure 1. As expected, modifier C displays an additional broad low-field resonance at 13.01 ppm. Therefore, the spectra are according to the molecular structures described in Scheme 1.

## 3. Holographic Set-Up

The samples prepared as described in Section 2 were exposed to a laser beam (λ = 532 nm) in a holographic set-up in order to record a diffraction grating in the photopolymer.

**Figure 2:**
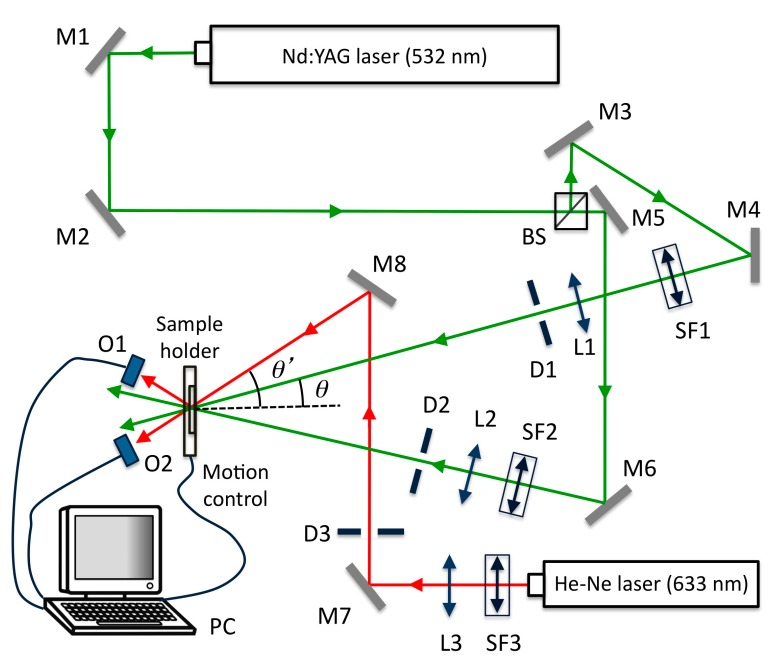
Holographic set-up. BS: Beamsplitter; Mi: Mirror; SFi: Spatial filter; Li: Lens; Di: Diaphragm; Oi: Optical power meter; PC: Data recorder.

We used the holographic set-up detailed in Figure 2 to study the behaviour of liquid crystal–photopolymer composites as a holographic recording material. A diode-pumped solid state laser (Spectra-Physics, Santa Clara, CA, USA) tuned at a wavelength of 532 nm with TE polarization was used to record unslanted diffraction gratings by means of continuous laser exposure. The laser beam was split into two secondary beams with an intensity ratio of 1:1. The diameter of these beams was increased to 1 cm by means of a pinhole and lenses, while spatial filtering was ensured. The object and reference beams were recombined at the sample at an angle of 16.0 degrees to the normal with an appropriate set of mirrors, and the obtained spatial frequency was 1036 lines/mm. The working intensity at 532 nm was 2 mW/cm^2^. The diffracted and transmitted intensities were monitored in real-time with a He-Ne laser positioned at Bragg’s angle (19.1°) with TE polarization and tuned to 632.8 nm, where the material does not polymerize. The recordings were made at 23 °C and the exposure time was adjusted to 40 s after several experiments to obtain the maximum diffraction efficiency without overmodulation.

After recording, the samples were exposed to 1 kHz square wave signal voltage by means of a Tektronic AFG3022B dual channel arbitrary function generator (Beaverton, OR, USA), an N4L voltage amplifier (Newtons4th Ltd, Leicester, UK), and an impedance control circuit designed in our laboratory to sustain a relatively high intensity avoiding the protection circuit of the amplifier. Root mean square (RMS) voltage and RMS current intensity were measured with Aim-TTi 1604 digital multimeters (Aim and Thurlby Thandar Instruments, Glebe Rd, Huntingdon, Cambridgeshire, UK). The diffracted light intensity as a function of the RMS voltage was monitored with the He-Ne laser.

In this kind of photopolymer, a photopolymerization reaction takes place in the exposed zones of the diffraction grating, and a highly cross-linked polymer network is generated. During the PIPS, the liquid crystal molecules diffuse to the unexposed region where they remain as droplets [3].

## 4. Results

### 4.1. Variation of the Electric Current with the Voltage

**Figure 3:**
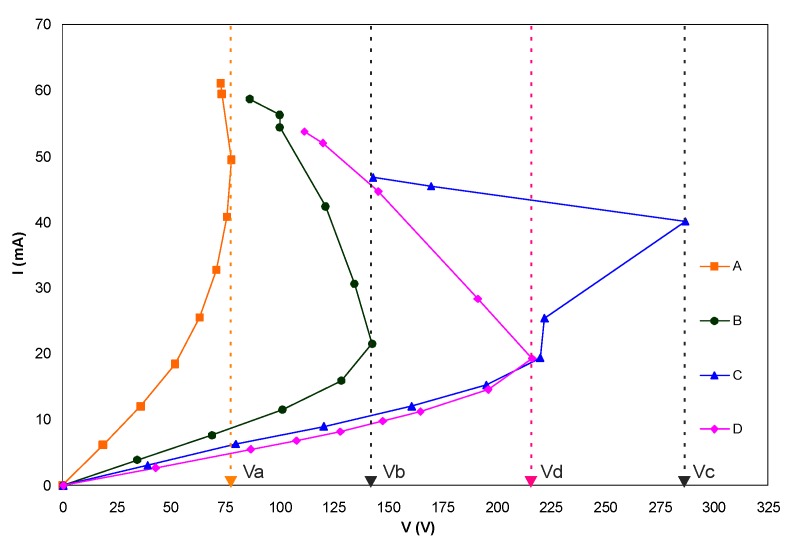
Current intensity versus voltage for samples A, B, C, and D.

The variation of the electric current passing through the sample with the voltage is shown in Figure 3. Current intensity (*I*) and voltage (*V*) are RMS values. Curves A, B, and C are samples with the modifiers shown in Scheme 1. The curve D corresponds to the sample without modifier.

We can see that the electric current passing through the samples increases with the applied voltage until reaching maximum voltages *V*_a_, *V*_b_, *V*_c_, and *V*_d_ respectively. From this point, the current intensity increases and the voltage decreases or remains approximately constant. It is important to highlight the differences in the maximum voltage obtained depending on the modifier introduced. Thus, *V*_a_ = 78 V, *V*_b_ = 143 V, *V*_c_ = 287 V, and *V*_d_ = 217 V. Taking account of the maximum voltage for the sample D without modifier (*V*_d_), the incorporation of modifiers to the photopolymer may increase (*V*_c_) or decrease this value (*V*_a_, *V*_b_). 

This result shows that the modifiers affect the electric behavior of the samples. A possible explanation related to the mode of action of the Schiff’s base additives focuses on the molecular structure of these substances and how they could help to increase the electric current through the sample. The modifiers have a molecular structure with delocalized π-electrons which can move from one end of the molecule to another, favouring the electric current through the sample. This capability is modulated by the presence of groups with donor effect (+ sign) or acceptor (− sign) of electrons, and this effect may be inductive (i) and/or mesomeric (m). This data are included in Table 2 for each modifier [16].

Modifier A with lower maximum voltage has two opposite MeO– groups sending additional π-electrons to the center of the molecule. Modifier C with higher maximum voltage has an HO– group sending additional π-electrons to one end of the molecule, making a polar head close to Cl atom.

### 4.2. Variation of the Diffracted Light Intensity with the Voltage

**Figure 4:**
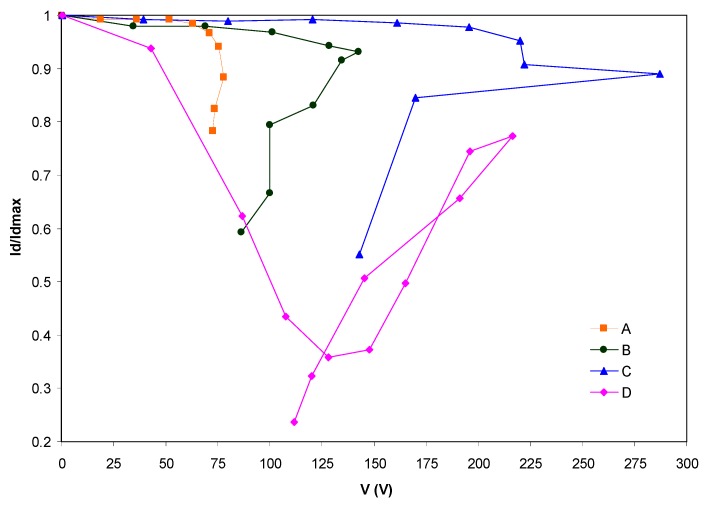
Diffracted light intensity divided by the maximum diffracted light intensity versus voltage.

Figure 4 shows the diffracted light intensity divided by the maximum diffracted light intensity (*I*_d_/*I*_dmax_) versus voltage for each sample.

The graph for sample D without modifier has the expected behavior for an H-PDLC device in a practical working range between 0 and 128 V. We increased the voltage for sample D, working out of this range to compare the result with samples A, B, and C.

For samples A, B, and C, it can be seen that the modifiers change the shape of the *I*_d_/*I*_dmax_ graph versus voltage, increasing the threshold voltage and decreasing the working range of the device that now acts similarly to an on/off switch for the diffracted light. This implies that the LC molecules cannot be oriented with the electric field at low voltage, and this is due to the electric interaction of the modifiers.

The result shows that additives with a specific molecular structure can modify the behaviour of the LC molecules in an electric field. We have used only one concentration of modifier in each sample which is close to the maximum solubility of the substances, and we have obtained a behavior-type on/off switch with different voltages depending on the modifier.

For future work, it will be necessary to study if a different concentration of the modifiers changes the working range of the device and if it is possible to obtain a different behaviour from on/off response.

### 4.3. Reconstruction of the Holograms

Additionally, we have studied the influence of the modifiers in the recording of the diffraction grating. The holograms were reconstructed with the He-Ne laser, and the angular response was obtained using a rotating stage. Afterwards, the samples were exposed to incoherent white light (1100 lx, 15 min) in order to stabilize the hologram, and they were reconstructed again.

**Figure 5:**
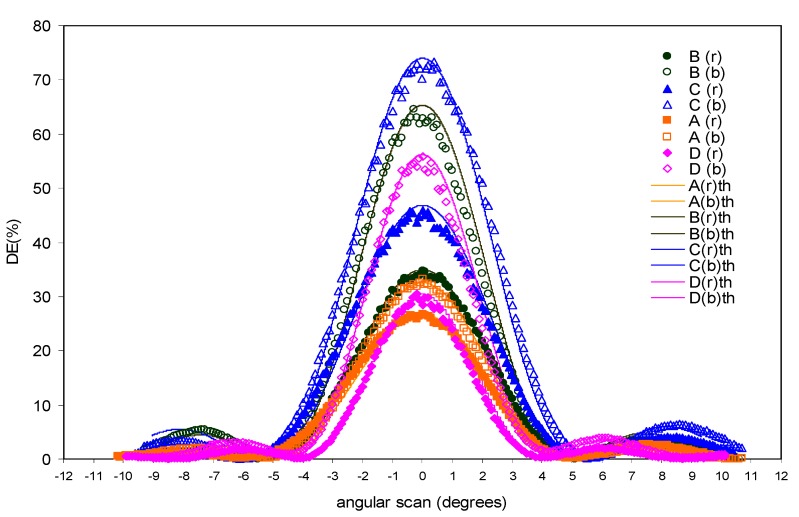
Experimental diffraction efficiency (DE) versus angular scan for photopolymers after recording: A(r), B(r), C(r), D(r), and after bleaching: A(b), B(b), C(b), D(b). The solid lines: A(r)th, B(r)th, C(r)th, D(r)th, and A(b)th, B(b)th, C(b)th, D(b)th show the theoretical fitting after recording and after bleaching, respectively.

Figure 5 shows the reconstruction of the hologram after recording (graphs r) and after the bleaching process (graphs b). The curves A(r), B(r), and C(r) with modifiers have the same shape as photopolymer D(r) without modifier. Therefore, the Schiff’s base additives do not stop the polymerization process.

It is important to highlight that the curves obtained after recording have different maximum diffraction efficiency (*DE*_max_): A(r) = 27%, B(r) = 35%, C(r) = 46%, D(r) = 30%. These differences are increased for the curves obtained after the stabilization process: A(b) = 33%, B(b) = 63%, C(b) = 73%, D(b) = 56%. C(b) obtains a relatively high *DE*_max_, and A(b) obtains a relatively low *DE*_max_. The order of the *DE*_max_ values is A < D < B < C for both curves r and b. This implies that the differences were initially produced during the hologram recording.

The diffraction gratings stored in the samples are phase and volume gratings, and the *DE*_max_ is related to maximum index modulation and thickness according to Kogelnik coupled wave theory [17].

(1) $$DE\max=\Gamma \sin^{2}\frac{\pi n_{1}{\left(t\right)}_{\max}d}{\lambda \prime \cos\theta_{i}\prime }$$

In Equation (1), Г is the absorption, diffusion, and reflection losses factor; θ_i_’ is the reconstruction beam angle (Figure 2), measured into the material, which can be obtained with refraction Snell’s law; λ’ is the reconstruction beam wavelength; *n*_1_(*t*)_max_ is the maximum refractive index modulation; and *d* is the diffraction grating thickness.

*DE*_max_ of each curve in Figure 5 is related to the product *n*_1_(*t*)_max_·*d* with the other factors approximately constant. The experimental data are fitted by an algorithm developed by our research team based on the rigorous coupled wave theory (RCW) [18,19]. The parameters obtained are included in Table 3.

Theoretically, according to Equation (1), the parameter d could be as important as *n*_1_(*t*)_max_ in order to obtain the *DE*_max_ value of an individual sample. For this set of samples, the data obtained from Table 3 (*d* and *n*_1_(*t*)_max_) suggest that the contribution of d has less influence than n_1_(t)_max_ in the differences of DEmax (th) values between samples. This can be easily seen experimentally with the width of the angular response curves (Figure 5), because it is related to d. When the width of the curve is large, the grating thickness d is small, and vice versa. The angular interval of the curves (r) measured at full width at half maximum (FWHM) is the next: A(r) = 5°, B(r) = 5°, C(r) = 5°, D(r) = 4°. There are no differences between curves A(r), B(r),and C(r), and the difference with curve D(r) is only one degree. Therefore, the FWHM is not responsible of the differences in *DE*_max_ values according to Equation (1). 

Therefore, we can conclude that the differences in the experimental *DE*_max_ values are directly related to the maximum refractive index modulation obtained by each formulation. For the set of samples, the influence of *d* or Г on the variations of *DE*_max_ is very small related to the refractive index modulation. 

The spatial modulation of the LC is the main mechanism implied in the refractive index modulation of this kind of photopolymer. Therefore, the result obtained suggests that the LC content in dark zones after recording is different for each photopolymer. Thus, the LC content in dark zones would be relatively high for photopolymer C and relatively low for photopolymer A. This difference is provided by the different additives present in each formulation. 

In-depth study and the use of analytical techniques are necessary in order to know why specifically the modifiers change the LC content in dark zones; i.e., the LC concentration spatial modulation in a different way depending on its molecular structure. The size of the modifiers is similar to those of the LC, and therefore they can influence the diffusion processes during de photopolymerization. The differences in the physicochemical characteristics of the modifiers could modify the diffusion processes of the LC molecules during the PIPS in a different way. If we see the molecular structures in Scheme 1, modifier A is an ether and modifier C is a halogenated substance. Both substances are chemically different from B. Therefore, this would change the number of LC molecules diffused to the dark zones and also perhaps the stacking or size distribution of the LC droplets. The composition of the LC droplets could also be different in the presence of modifiers, and these could influence the refractive index of the dark zones.

**Figure 6:**
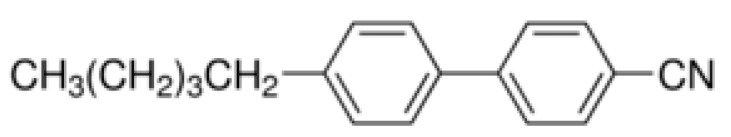
4′-Pentyl-4-biphenylcarbonitrile is representative of the liquid crystal mixture.

We can see the structure of the LC molecule in Figure 6. The CN substituent has effect −i, −m, and the molecule has delocalized π-electrons and a polar head close to NC– group. We can see in Scheme 1 that the modifier C can be represented in the same way. The Cl atom has an electronegativity of 3.0 in the Pauling’s scale, equal to the nitrogen atom of the NC– in the LC.

This structural similarity of the modifier C with the LC can be responsible for the highest *DE*_max_ obtained for the polymer with this additive. On the contrary, modifier A obtains the lowest *DE*_max_. The chemical structure of this substance is different from those of LC because it has two MeO– groups in opposite ends. Modifier B without polar head but with electronic delocalization and photopolymer D without additive obtain *DE*_max_ between A and C.

## 5. Conclusions

We have observed that the addition of Schiff’s bases with specific π-electron system to the formulation of H-PDLC composites affects the electric current passing through the sample when a variable electric field is applied. A high or low maximum voltage is obtained depending on the specific molecule used as additive. We have obtained a correlation between the maximum voltage and the inductive or mesomeric effect of the substituents in the additives. On the contrary, the shape of the *I*_d_/*I*_dmax_ graph versus voltage is modified and the response of the H-PDLC device is changed to an on/off switch, decreasing its working range. It is necessary to study if the modification of the additive concentrations could improve this aspect. The result also suggests a possible work path by means of the introduction of specific substituents with inductive/mesomeric effect in the liquid crystal molecule itself. For both cases, additional studies are necessary.

We have also obtained that these additives influence the maximum diffraction efficiency reached after holographic recording and after stabilization process. This is related to differences in the diffusion processes of the LC molecules during the PIPS. The result is very interesting in order to improve the response for static holograms. In order to explain this behaviour, a more detailed study is necessary. A hypothesis is related to the different chemical characteristics of the additives depending on the type of substituent. Modifier C is a halogenated substance, and obtains a relatively high maximum diffraction efficiency. On the contrary, modifier A is an ether and obtains relatively low maximum diffraction efficiency.

## Figures and Tables

**Table 1:** Composition of the holographic photopolymer. DPHPA: dipentaerythritol penta/hexa-acrylate; LC: liquid crystal; YEt: ethyl eosin; NPG: *N*-phenyl glycine; OA: octanoic acid; NMP: *N*-methyl-2-pyrrolidone.

Component	Concentration (wt %)
DPHPA	43.00
LC	29.50
YEt	0.04
NPG	0.36
OA	8.50
NMP	18.60

**Table 2:** Effect produced in the additive molecule by the substituents.

Sample	Modifier	Substituents Main Effect	Effect Produced in the Modifier Molecule
A	A	MeO–: +m	Two opposite MeO– groups send π-electrons to the center of the molecule
B	B	Without i/m effect	π-electron mobility
C	C	HO–: +m	HO– group sends π-electrons to one end of the molecule making a polar head close to Cl atom
		Cl–: −i	
D	Without additive	Without effect	Without effect

**Table 3:** Parameters obtained with rigorous coupled wave theory (RCW).

Sample	*d*	Г	*n*_1_(*t*)_max_	*DE*_max_ (th)
	(μm)			(%)
A(r)	15.5	0.155	0.0075	27
B(r)	16.0	0.192	0.0087	35
C(r)	14.8	0.148	0.0110	47
D(r)	18.0	0.180	0.0105	30
A(b)	16.0	0.224	0.0085	33
B(b)	16.0	0.160	0.0085	65
C(b)	14.5	0.145	0.0160	74
D(b)	19.8	0.198	0.0065	56
